# Dynamics of receptor and protein transducer homodimerisation

**DOI:** 10.1186/1752-0509-2-92

**Published:** 2008-10-31

**Authors:** Julio Vera, Thomas Millat, Walter Kolch, Olaf Wolkenhauer

**Affiliations:** 1 University of Rostock, 18051 Rostock, Germany; 2 The Beatson Institute for Cancer Research, Glasgow G61 1BD, UK; 3 University of Glasgow, Sir Henry Wellcome Functional Genomics Facility, Glasgow, G12 8QQ, UK

## Abstract

**Background:**

Signalling pathways are complex systems in which not only simple monomeric molecules interact, but also more complex structures that include constitutive or induced protein assemblies. In particular, the hetero-and homo-dimerisation of proteins is a commonly encountered motif in signalling pathways. Several authors have suggested in recent times that dimerisation relates to a series of physical and biological outcomes used by the cell in the regulation of signal transduction.

**Results:**

In this paper we investigate the role of homodimerisation in receptor-protein transducer interactions. Towards this end, mathematical modelling is used to analyse the features of such kind of interactions and to predict the behaviour of the system under different experimental conditions. A kinetic model in which the interaction between homodimers provokes a dual mechanism of activation (single and double protein transducer activation at the same time) is proposed. In addition, we analyse under which conditions the use of a power-law representation for the system is useful. Furthermore, we investigate the dynamical consequences of this dual mechanism and compare the performance of the system in different simulated experimental conditions.

**Conclusion:**

The analysis of our mathematical model suggests that in receptor-protein interacting systems with dual mechanism there may be a shift between double and single activation in a way that intense double protein transducer activation could initiate and dominate the signal in the short term (getting a fast intense signal), while single protein activation could control the system in the medium and long term (when input signal is weaker and decreases slowly). Our investigation suggests that homodimerisation and oligomerisation are mechanisms used to enhance and regulate the dynamic properties of the initial steps in signalling pathways.

## Background

The processing of information in living cells is carried out by signal transduction pathways [1]. Through the binding of external ligands to extracellular receptors, the cell can receive signals from its environment and transfer information into the cell. This information flow is regulated, amplified or modulated by different feedback mechanisms and interactions with other pathways (crosstalk). Moreover, signalling pathways are complex systems in which not only simple monomeric molecules interact but also more complex structures that include constitutive or induced protein assemblies [2-4]. In particular, the hetero- and homo-dimerisation of proteins is a commonly encountered motif in signalling pathways.

In Klemm [5] the role of dimerisation as a regulatory mechanism in signal transduction is analysed and discussed. Dimerisation is defined as an interaction producing a protein-protein complex composed of two subunits, either identical (homodimerisation) or non-identical (heterodimerisation). The authors argue that dimerisation relates to a series of physical and biological outcomes used by the cell in the regulation of signal transduction. The biophysical outcomes referred to facilitation of proximity and orientation in protein interaction, differential regulation through heterodimerisation, emergence of spatio-temporal boundaries, enhanced specificity and regulation of monomer-to-dimer transitions. The role of homodimeric receptors in the activation and dimerisation of intermediate proteins and transcription factors has been already described in the literature [6-8].

A well-studied example are the JAK/STAT signalling pathways [9]. In case of the JAK2/STAT5 signalling pathway, the Epo receptor is a preformed inactive dimer in the plasma membrane [10,11]. The binding of Epo results in the activation of the JAK2 kinase and subsequent phosphorylation of the cytosolic domain of each Epo receptor monomeric subunit. STAT5 proteins bind to the tyrosine phosphorylated Epo receptor and gets phosphorylation. Afterwards, they dimerised and translocate to the nucleus. The spatial conformation of the receptor as a dimer seems to indicate that each activated Epo receptor monomer could phosphorylate simultaneously at least one STAT5 molecule. The correspondence between the existence of a homodimer activated receptor and the activation of a homodimer transduction protein suggests a possible complex underlying molecular mechanism for the activation and dimerisation process. Similar behaviour has been shown in JAK/STAT pathways [12] and other signalling pathways [13-18], suggesting that this homodimer-homodimer interaction could constitute a more general pattern in cell signalling systems.

The purpose of this work is to use mathematical modelling to suggest mechanisms of interaction by which this homodimer-homodimer interaction can occur. In our work, two mathematical modelling frameworks are used and compared. We furthermore investigate the dynamical consequences of the interaction mechanisms suggested and propose general features of an experimental design to discriminate between the different mechanisms.

## Results and discussion

### Mechanistic modelling

In this paper we support the hypothesis that in the transduction of signals via homodimeric proteins, the dimeric nature of the receptor plays an essential role in the fast response of the biological system. The surface density of many plasma membrane receptors tends to be very low [19]. For a low density of receptors at the plasma membrane, active mechanisms for the homodimerisation of the cytosolic interacting proteins are required to boost the intracellular response of the system to external stimuli. Towards this end, we propose that the dimeric structure of the receptor allows not only the simple activation of a monomer binding protein per receptor but also a simultaneous coordinated activation of two monomers of the binding protein, one at each subunit of the receptor. The case in which we consider the single interaction between one subunit of the receptor and a monomer of the protein transducer, we call "single protein activation process" with respect to the number of receptor-protein interactions. In contrast, two subunits of the same receptor interacting simultaneously with two different monomers are referred to as a "double protein activation process". In the following we are going to assume that higher-order processes, allowing for simultaneous activation of two or more protein monomers at the same receptor monomer, are not significant. Several studies with the PDGF receptor in which all tyrosine phosphorylation sites were mutated individually or in combination showed that the signalling was unchanged when redundant sites were knocked out, suggesting that this assumption is in principle meaningful [20].

The mathematical framework considered describes the temporal evolution of protein concentrations with coupled ordinary differential equations. The rate of change of molecular species X_*i *_(number of activated or inactivated protein transducer and receptors) is expressed as:

(1) $$\frac{dX_{i}}{dt}=\sum_{j}\sigma_{ij}\cdot \gamma_{j}\prod_{k}X_{k}^{g_{jk}},$$

where *γ*_*j *_are rate coefficients, *g*_*jk *_kinetic orders and *σ*_*ij *_the stoichiometric coefficients. The stoichiometric coefficient *σ*_*ij *_is positive for products and negative for reactants and describes how many molecules of X_*i *_are converted in the considered reaction. The rate coefficients contain information about the physical properties of the reaction, like activation energies and internal states [21]. If we assume that environmental conditions, like temperature, pressure and pH-value do not vary over time, *γ*_*j *_is a rate constant. The interpretation of the kinetic order *g*_*jk *_depends on the chosen level of description for the biochemical network. When (1) is derived within the framework of statistical physics and the complete reaction mechanism is known and considered, the coefficient *g*_*jk *_has a clear mechanistic meaning and defines the number of molecules of species *X*_*i *_involved on the considered reaction. The kinetic order has in this case a positive integer value. In contrast, if we aggregate elementary reaction steps into a combined power-law expression, with the same form of (1), the interpretation of the coefficient *g*_*jk *_changes and they can take non-integer values [22-25]. This latter case is investigated in the next section.

For the purpose of our investigation, we consider the generic case of a diffusive signalling protein (for simplicity, named *protein*, *P*, in our discussion), which is activated and dimerised after the interaction with a homodimeric activated receptor (named *receptor*, R*). In order to focus the investigation on general design principles underlying the homodimer receptor-homodimer transducer protein class of interactions, we simplify the mathematical modelling of the system, deliberately neglecting details that account for differences in the class. The procedure is similar to the one used in [26].

**Figure 1:**
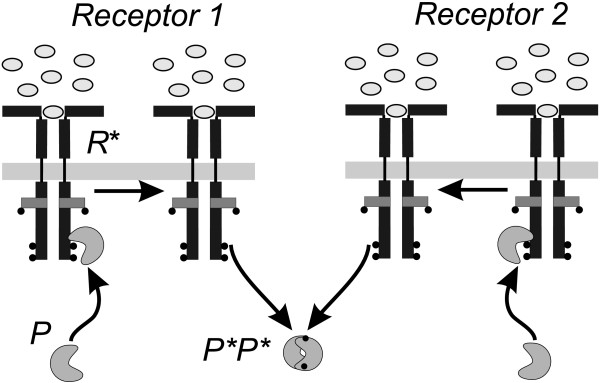
**Activation of the signal transduction protein**. The activation of the signal transduction protein *P *in the single protein activation process. Two protein monomers (*P*) bind independently to two different activated receptors (R*). After activation at the receptors the modified monomers (P*) are released to the cytosol where they form the activated dimer (P*P*). This dimer then activates the subsequent levels of the pathway.

We propose a model with single protein activation of the protein dimer as shown schematically in Figure 1. In this process, two protein monomers, *P*, bind to two independent activated receptors, R*. As a consequence, the monomers becomes activated, P*, and will be released back into the cytoplasm. Finally, the monomers form an activated homodimer, (P*P*), which transduces the signal downstream. The activation of the dimer can be expressed by the following stoichiometric equations:

(2a) $${\text{R}}^{*}+\text{P}\overset{k_{1}}{\to }{\text{R}}^{*}+{\text{P}}^{*}$$

(2b) $${\text{R}}^{*}+\text{P}\overset{k_{1}}{\to }{\text{R}}^{*}+{\text{P}}^{*}$$

(2c) $${\text{2P}}^{*}\overset{k_{2}}{\to }{\text{(P}}^{*}{\text{P}}^{*}).$$

Equations (2a) and (2b) describe the independent monomer activation, whereas (2c) describes the formation of the dimer in the cytosol. In addition to its concentration, each reaction is determined by a rate constant *k*_*i*_, encapsulating physical information about the underlying biochemical reaction. For simplification purposes we apply additional assumptions. Firstly, since in our simplified model there is no production or additional recruitment of protein transducer *P*, there is an intrinsic conservation for *P *and P* in the model. In addition, we assume that the receptor activity remains constant as consequence of a constant external signal [27]. The dynamic control of receptor activity/concentration and the recruitment/recycling of protein transducers [28] are important features in signalling systems that must be considered when modelling specific signalling pathways, but can be neglected here for the purpose of generalisation. Finally, we furthermore assume that in the class of interactions investigated, the intermediate state R*P cannot go down a monomeric activation route [28].

**Figure 2:**
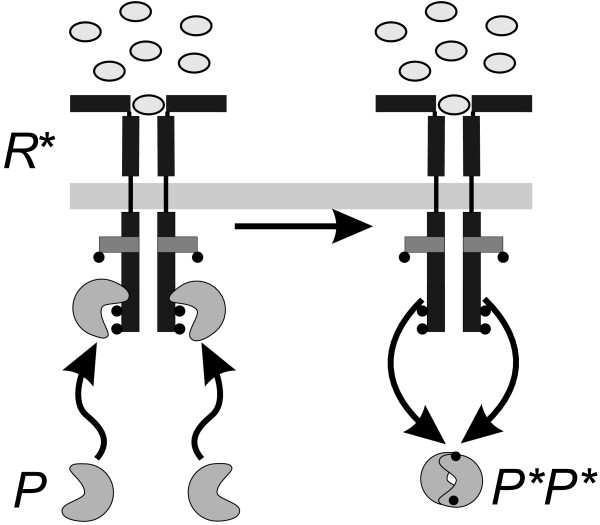
**Double activation process**. Schematic representation of the double protein activation process. Due to the dimeric structure of the receptor *R*, two inactive monomers can bind simultaneously to each active subunit. Each monomer becomes phosphorylated and releases to the cytosol. There, the activated monomers bind to a dimer, which translocates into the nucleus or activates the downstreamn signalling.

The stoichiometric representation (2) can be transformed into a set of coupled differential equations. The rate of change of the activated monomers is then

(3) $$\frac{dP_{1}^{*}}{dt}=2k_{1}\cdot R^{*}\cdot P-2k_{2}\cdot {\left(P_{1}^{*}\right)}^{2},$$

where the first term on the right-hand side corresponds to the activation of the monomers and the second term to the dimerisation. The activation of the monomeric protein depends linearly on the concentration of the protein and the activated receptors. The subscript of *P *on the left-hand side denotes this fact. Apart from the above single activation process, we also consider a double protein activation. Here, two monomers bind to the same activated receptor, as shown in Figure 2. The stoichiometric formula of the double protein activation is

(4a) $${\text{R}}^{*}+2\text{P}\overset{k_{3}}{\to }{\text{R}}^{*}+2{\text{P}}^{*}$$

(4b) $$2{\text{P}}^{*}\overset{k_{4}}{\to }{\text{(P}}^{*}{\text{P}}^{*}),$$

where the first term describes the double protein activation process and the second the dimer formation. In contrast to (2a) and (2b), the molecularity of protein *P *in the process of phosphorylation is now two instead of one. The process of dimerisation has the same structure as in the single protein activation process.

Because we focus on the consequences of the combination of single protein activation and double protein activation mechanism, we neglect some internal details of the double mechanisms and merge it into a single expression (For more details [see Additional file 1]). The temporal changes of the activated protein concentration are given now as

(5) $$\frac{dP_{2}^{*}}{dt}=2k_{3}\cdot R^{*}\cdot P^{2}-2k_{4}\cdot {\left(P_{2}^{*}\right)}^{2}.$$

In analogy to stoichiometric equation (4), the phosphorylation of the protein is a second-order process with respect to its inactive form *P*. The subscript 2 in (5) denotes the involved double protein activation process in the activation of the protein. We notice that for simplicity we used reduced (simplified) representations for the processes to focus on the effects of the combined activation mechanism suggested in this work. The modeling of specific cellular systems may require a more detailed description with respect to the biochemical interactions. In the following we assign for simplicity the same value to the rate constants *k*_2 _and *k*_4 _and focuss our attention on the different mechanisms of activation. If we assume that both activation mechanisms occur simultaneously and that the phosphorylated monomers are indistinguishable for the cell and for the experiments performed, the total concentration of active monomeric proteins is obtained as the sum of both 'species'

(6) $$P_{\text{T}}^{*}=P_{1}^{*}+P_{2}^{*}.$$

Consequently, the change of concentration of the active form P* results from a summation of both contributing activation processes

(7a) $$\begin{array}{ccc} \frac{dP_{\text{T}}^{*}}{dt} & = & \frac{dP_{1}^{*}}{dt}+\frac{dP_{2}^{*}}{dt} \end{array}$$

(7b) $$\begin{array}{lll} \frac{dP^{*}}{dt} & = & \left[2k_{1}\cdot R^{*}\cdot P-2k_{2}\cdot {\left(P^{*}\right)}^{2}\right]\\ & = & +[2k_{3}\cdot R^{*}\cdot P^{2}-2k_{2}\cdot {\left(P^{*}\right)}^{2}]. \end{array}$$

After some algebraic transformation a simplification arises:

(8) $$\frac{dP^{*}}{dt}=2k_{1}\cdot R^{*}\cdot P\left[1+\frac{k_{3}}{k_{1}}P\right]-4k_{2}\cdot {\left(P^{*}\right)}^{2},$$

where we arrange the contributions of the considered activation processes and dimerisation into separated terms. As expected from our discussion above the rate law (8) contains a combination of the single and double protein activation processes. However, from the above rate law we can easily derive limiting cases, where one of the considered processes is dominant. To this end, we analyse the expression in the brackets of Eq. (8). On the one hand, if we have

$$\begin{array}{ccc} 1\gg \frac{k_{3}P}{k_{1}} & \text{or} & k_{1}\gg k_{3}P \end{array}$$

the double protein activation is negligible in comparison to the single protein activation. The apparent kinetic order of *P *in the rate equation tends to *g*_1 _= 1. If we have

$$\begin{array}{ccc} 1\ll \frac{k_{3}P}{k_{1}} & \text{or} & k_{1}\ll k_{3}P \end{array}$$

then the double protein activation is dominant. The apparent kinetic order tends to the limiting value of *g*_2 _= 2. Between the limiting cases discussed above, both activation mechanisms contribute to the dynamics and the system is not simply single or double protein activation. An elementary, positive and integer, kinetic order cannot be assigned to the overall reaction and an apparent positive non-integer kinetic order occurs. The origin of this apparent kinetic order as well as its dependence on the kinetic constants are discussed in the section below.

### Power-law modelling

An experiment measuring the concentration of the protein, active and inactive receptors and/or protein activated dimers, cannot distinguish between active monomers which are produced in a single or double protein activation process. To distinguish the different activation mechanisms, an indirect method is required, another possibility is to investigate the structure of the protein. However, the detection of two binding sites at the same receptor, does not guarantee that both sites are used simultaneously nor that this is necessarily an effective activation process.

In order to investigate the possibility of two different and simultaneously acting phosphorylation mechanisms further, we aggregate both processes introduced in the above section into a single contribution. The order of the receptor *R *in the activation process is one, as in the mechanistic model (8). However, the contribution of protein *P *is a combination of the considered single and double activation processes. Since both activation mechanisms described by *k*_1 _and *k*_3 _are indistinguishable, the estimation of these kinetic parameters generates identifiability issues. If we consider a situation in which both processes cannot be distinguished, a feasible way to reproduce this complexity is the use of a power-law representation, allowing for non-integer kinetic orders. As discussed in [25], power-law models are useful for modelling cellular signalling when the exact reaction mechanism is unknown or if experimental data are not sufficient. For an example in cell signalling we refer to [29]: in that work, quantitative time course data were used to identify a power-law model. If we used a power-law term, the production term (phosphorylation of *P*) of the new rate equation has the following structure:

(9) *V*(R*, *P*) ≈ *γ*_1_·*R*·*P*^*g*^.

The apparent kinetic order *g*, which describes the role of the protein, has now a non-integer value between one and two. The specific value of *g *depends on the rate constants *k*_1_, *k*_3 _and the concentration of the inactive protein *P *as shown in the subsection "Mechanistic modelling". We note that this apparent kinetic order is only a mathematical analogue to the kinetic order of elementary reactions. It does not provide detailed information about the underlying mechanisms and hence, it cannot be interpreted in the same way as the kinetic order of an elementary reaction scheme. In addition to the apparent kinetic order *g*, we also introduce an apparent kinetic constant *γ*_1 _in the power-law representation (9). Due to the aggregation of production terms in (9), this apparent kinetic constant does not coincide with the kinetic constant in the mechanistic rate law (8). Nevertheless, it depends on the kinetic constants *k*_1 _and *k*_3 _but also on the ratio of efficiency of single and double protein activation processes.

**Figure 3:**
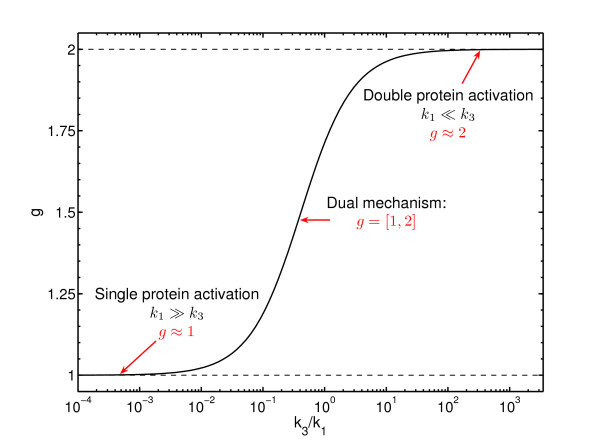
**Apparent kinetic order *g***. The apparent kinetic order *g *for production term (9) as a function of the ratio *k*_3_/*k*_1_. The value of the kinetic order changes from *g *= 1 in the limit *k*_1 _≫ *k*_3 _to a value *g *= 2 for *k*_1 _≪ *k*_3_. The approximated kinetic order was calculated using the approach described in [30], assuming an interval of feasible values for *P *[0.0; 4.0], where 1.0 represents a normal level of expression and 4.0 intense overexpression.

In the considered case, the differences in the efficiency of the considered mechanisms are determined by the production terms of single (3) and double protein activation processes (5). In our analysis we neglect the common contributions of the activated receptor concentration, R* and constant prefactors in both terms. As a consequence, the ratio of efficiency is mainly determined by the ratio (*k*_3_) = *k*_1_. A change in this ratio will change the apparent kinetic order *g *(See Figure 3). We used the method proposed in [30]. As expected, the apparent kinetic order *g *in Eq. (9) changes its value if the ratio of the phosphorylation mechanisms is modified. It increases if the contribution of the double activation process is augmented in comparison to the single activation process. As discussed in the subsection "Mechanistic modelling", the kinetic order is limited by the value *g *= 2, when the complete phosphorylation of *P *is realised by the double protein activation process. The lower limit is *g *= 1, when the single activation process is dominant.

In the general case and under the assumption that we do not know the exact relation between the underlying (indistinguishable) processes (encoded by the ratio *k*_3_/*k*_1_) the power-law representation allows us to reproduce essential dynamical properties of the system, regardless whether the ratio is low, medium or high [see Additional File 1]. A power-law model can therefore be used to elucidate the nature of the underlying mechanism. An estimated value for the kinetic order *g *near one would suggest that the single protein activation mechanism is the principal or unique contributor to the activation process while a value near two would indicate the relevance of the double protein activation process. An intermediate value would indicate that the mechanism has a dual nature as described in the previous section.

### Dynamical Consequences of the Dual Mechanism

**Figure 4:**
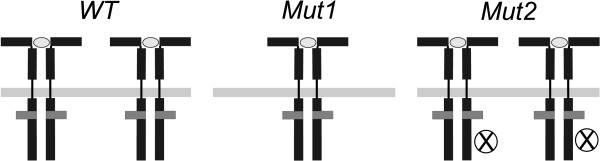
**Experimental design**. Experiment proposed to elucidate the activation of homodimer proteins by homodimer receptors. Schematic representation of mutant cells (Mut1 and Mut2) with respect to the wild-type cells (WT).

**Figure 5:**
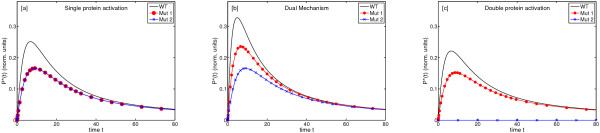
**Time courses**. Hypothetical time courses of the concentration of active protein P* for wild-type (WT) and mutated cells (Mut1, Mut2). The kinetic parameters *k*_1 _and *k*_3 _are the same for the three configurations (*k*_3_/*k*_1 _= 0.5). The initial conditions are: *P*(0) = 1; P* = 0. Figure 5 [a]: response of both mutants when the system is supposed to have only single protein activation. Figure 5 [b]: response of both mutants when a dual mechanism of activation is considered. Figure 5 [c]: response when the mechanism of activation is double protein activation. The configuration of the system for wild-type cells implies an initial intensity for the activated receptor *R*(0) = 1, while in mutant cells *R*(0) = 0.5.

In previous sections we assumed that protein *P *can be activated by two mechanisms of different order. In the present section we discuss how the dynamics of the system change under different experimental conditions: a) when the activation of a homodimer protein by a homodimer receptor is proceeded by a dual mechanism as considered above or b) with a simple mechanism, of single or double activation. Towards this end, we compare the behaviour of wild-type cells to the response of two mutants, as shown in Figure 4. In the first case (Mut1), the dynamics of the receptor recruitment are altered, reducing the amount of receptors available for activation at the plasma membrane to half. In the second case (Mut2), one of the monomers in the receptors is constitutively blocked and therefore unable to activate the protein *P*. The feasibility of such experiments was demonstrated by Behrmann and collaborators [31] in a similar system where the activation and dimerisation of STAT1 by the interleukin 5 receptor (IL-5R) was investigated. Other aspects of the cells remain unchanged with respect to the wild-type cells, including the total available concentration of protein *P*. In the simulations, the time-dependent fraction of activated homodimeric proteins (P*) in the three types of cells (WT, Mut1 and Mut2) is measured after equivalent stimulation. The modifications change the contribution of the single and double activation processes. In the first case (Mut1), both activation mechanisms, single and double, are still possible but the number of binding sites in the receptors is half of the amount existing in wild-type cells. This decrease of the ratio of receptors and inactive protein *P *increases the probability of the double protein activation process. Hence, the contribution of this double protein activation to the net activation rate is increased. In the second case (Mut2), the number of binding sites in the receptors is also half of the amount existing in wild-type cells but only the single protein activation process is possible. In Figures 5[a–c] we compare the response of Mut1, Mut2 and WT to the same stimulation experiment in three different cases. Figure 5[a] shows the results for a receptor-protein system where only the single protein activation is possible. The response of the system to the stimulation when the dual mechanism is feasible is represented in Figure 5[b]. Finally, in Figure 5[c] the dynamics of the system are shown if only the double protein activation process is feasible.

The comparison of the three situations shows clear differences between the different scenarios. If the system allows only the single protein activation process for *P*, the response of the system in both mutants would be indistinguishable (Figure 5[a]). In contrast, if the system allows the dual mechanism, single and double protein activation, the response is different for Mut1 and Mut2 (Figure 5[b]). Moreover, on the assumption of an intermediate value for the ratio *k*_3 _*P*/*k*_1 _in this case, the differences in the response between wild-type and mutant cells are more significant (Figure 5[b]). Finally, if the activation mechanism is a double protein activation process, only Mut1 should produce a significant signal after stimulation (Figure 5[c]).

A system, where the stimulation with the same input signal produces identical response in both mutants, would not present the dual mechanism of activation but only the single activation. A system where the responses for the same input signal are differentiated in both mutants would present the dual mechanism of activation. Finally, a system in which the stimulation of the system produces a signal for the Mut1 but not for the Mut2 (where any double protein activation process is intentionally blocked) is a system where only the double protein activation process is possible.

**Figure 6:**
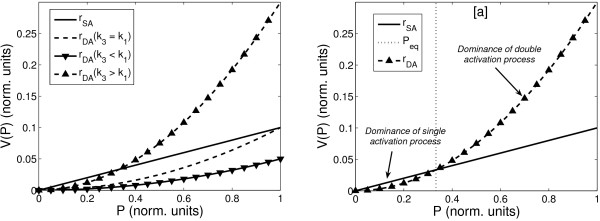
**Comparison of single and double activation**. Comparison between single protein (*r*_SA_) and double protein (*r*_DA_) activation rates for the interval of feasible values of inactive protein *P*. Normalised units were used in this comparison (*P *= 1 is total amount of available protein). Figure 6 [a]: Comparison of the three possible cases. In solid black we represents the value of the single activation signal rate (*r*_SA_) for *k*_1 _= 0.3 in the interval of feasible values of *P *(0.0 ≤ *P *≤ 1.0). The dashed line represents double activation rate (*r*_DA_) when both rate constants are identical (*k*_3 _= *k*_1_). Upward triangles represent the double activation rate when *k*_3 _> *k*_1 _and downward triangles when *k*_3 _<*k*_1_. Figure 6 [b]: Only with *k*_3 _> *k*_1 _the double protein activation dominates but for high protein concentrations. The concentration of *P *in which contributions from both single and dual activation signal rates are identical is called *P*_eq _= *k*_1_/*k*_3 _and is represented with a finely dashed line.

We now discuss under which dynamical conditions the dual mechanism emerge. In the following analysis we assume normalised units for the variables; for which a value equal to one represents the total amount of protein *P *in wild type cells. The most interesting behaviour emerges when the kinetic constant, characterising the double protein activation process, is higher than the one for the single protein activation process (*k*_3 _> *k*_1_) (Figure 6[a]). In this case, the signal rate associated to double protein activation (*r*_DA_) dominates for high concentrations of inactive protein *P *whereas single activation is more intense for low *P *values. Otherwise (*k*_3 _≤ *k*_1_), single activation rate (*r*_SA_) dominates over the whole interval of feasible concentrations for the protein (0.0–1.0). Furthermore, the importance of the double protein activation mechanism increases with the value of *k*_3 _from high to low concentrations of inactive protein.

In case of *k*_3 _> *k*_1_, the concentration of inactivate protein for which both signal rates contribute with an identical amount of signal (*k*_1_*P*_eq _= *k*_3_(*P*_eq_)^2^) is defined by the ratio between rate the constants *k*_1_/*k*_3 _(Figure 6[b]). The higher the value of this ratio is, the more reduced the effect of double activation in the dynamics of the system. On the other hand, reduced values of the ratio imply that double activation dominates even at much reduced concentrations of inactive protein. When the value of *k*_3 _increases the position of *P*_eq _is shifted to smaller values of *P*.

In order to establish the domain of values for the rate constants in which the mechanism is effectively dual, we fix an interval of feasible values for the parameter *k*_3 _with respect to *k*_1_. The minimum value for *k*_3 _is such that double protein activation rate contributes a 10% of the single activation signal for the maximum amount of inactive protein: *r*_DA_(*P *= 1) = 0.1·*r*_SA_(*P *= 1.0). Below this value for *k*_3_, the contribution of double activation (*r*_DA_) is negligible for any concentration of *P*. The maximum value of *k*_3 _is such that the double protein activation is dominant for values of *P *higher than 10% of the total amount: *P*_eq _= *k*_1_/*k*_3 _= 0.1. Lower values for *P*_eq _implies that single protein activation process does not contribute at any significant concentration of *P*. Thus, we define the interval of values for *k*_3 _in which the mechanism is dual like *k*_1_/10 ≤ *k*_3 _≤ 10*k*_1_. In this interval of values, the system presents a dual mechanism, with dominance of the single protein activation for low concentration of inactive protein and dominance of double protein activation for high values of *P*.

One of the dynamical consequences of this dual mechanism relates to the behaviour of the system during transient stimulation. Under certain circumstances there could be a switch between both mechanisms, with a dominance of the double protein activation in the short initial time (when the amount of inactive protein is high) and a take over the dynamics by the single protein activation during the medium-long term (when the amount of inactive protein is smaller).

This feature could be indeed reinforced by the effects that the asymmetric deactivation of the receptor by phosphatases and other signal terminators have in the dynamics of the system. In order to illustrate this idea, we assume that the deactivation of an activated receptor happens in two steps: first, one of the subunits of the receptor is deactivated by phosphatases and subsequently the other subunit is deactivated:

(10) $${\text{R}}^{*}{\text{R}}^{*}\overset{k_{\text{D}1}}{\to }{\text{R}}^{*}\text{R}\overset{k_{\text{D}2}}{\to }\text{RR}$$

For simplicity, we suppose that there is no cooperativity in the receptor deactivation process and therefore both deactivation rates are similar (*k*_D1 _= *k*_D2_). A fully activated receptor (R* R* is able to participate in either single or double protein activation whereas a partially activated receptor (R* R) can only participate in the single activation process. Indeed, the stoichiometry of the activation by the fully activated receptor is not the same for single and double protein activation: fully activated receptors have double activation sites for single activation processes. Considering all this, the equation describing the dynamics of protein activation becomes:

(11) $$\frac{dP^{*}}{dt}=2k_{1}\cdot (2\cdot R^{*}R^{*}+R^{*}R)\cdot P+2k_{3}\cdot R^{*}R^{*}\cdot P-4k_{2}\cdot {\left(P^{*}\right)}^{2},$$

**Figure 7:**
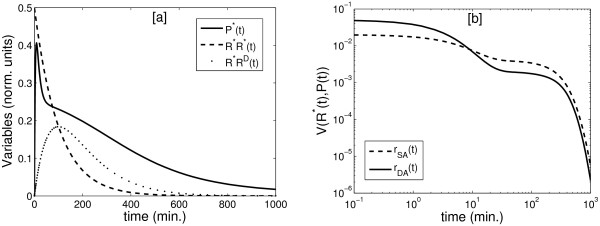
**Dynamic simulation**. Dynamical simulation of the system when dual mechanism and asymmetric deactivation of the receptor are assumed. The dynamics of the protein was complemented with a rate describing de deactivation and break down of the homodimeric protein P*P* ($r_{P^{*}P_{\text{deact}}^{*}}$ = *k*_4_·P*P*). Figure 7 [a]: Time course for the relevant variables of the system: monomeric activated protein (P*), fully activated receptor (R*R*) and partially activated receptor (R*R). Figure 7 [b]: Time course for single (*r*_SA_) and double (*r*_DA_) protein activation rates during the simulation. Initial conditions: *P*(0) = 1.0; P*(0) = 0.0; R*R*(0) = 0:5; R*R(0) = 0.0. Parameter values: *k*_1 _= 0.02; *k*_2 _= 0 > 05; *k*_3 _= 5*k_1 _= 0.1; *k*_4 _= 0.0125; *k*_D1 _= *k*_D2 _= 0.01.

We show the combined effect of the dual mechanism and the asymmetric deactivation of the receptor in Figure 7. In this simulation, the initially inactive system (*P*(0) = 1) is stimulated with a saturated transient stimulation of the receptors (all the available receptors are activated at the beginning of the stimulation: R*R*(0) = *R*_T_). In Figure 7[a] we represent the dynamics of the relevant variables of the system during the simulated experiment. We can see that the total available amount of receptors is fully activated at the beginning of the stimulation. The action of the phosphatases transform fully activated receptors into partially activated ones and from there provokes total deactivation of the receptors. Figure 7[b] compares the values of both single and double protein activation signal rates during the simulation. As we can see, in the very beginning of the signal (first ten minutes) the double activation mechanism is dominant and contributes almost all of the signal produced. After this initial period, the single activation signal rate becomes predominant in the medium-term (from ten to hundreds minutes) and continues until the termination of the signal. This suggests that in a dual mechanism there is a shift between both dynamics in a way that intense double protein activation initiates the signal and is essential in the short term to get a fast intense signal, while single protein activation contributes in the medium and long term and, when signal is weak and decreases slowly.

## Conclusion

In this paper, the possibility of a dual mechanism of activation for homodimer proteins by homodimer receptors is analysed. We propose a mechanistic model in which this peculiar interaction between homodimers provokes a dual mechanism of activation, which is simultaneously single and double protein activation process. Under the stated assumptions, the dual mechanism could appear essentially as either single or double activation, depending on the ratio between the rate constants of both process and the available amount of inactive protein. For a certain interval of values for these coefficients, the process can have an intermediate behaviour, which cannot be reduced to the single or double activation mechanism.

We further analysed a phenomenological power-law representation, which is able to adequately simulate the response of the system. We demonstrated that power-law representation is useful for cases where experimental data are available, but which are not sufficient to distinguish and characterise the reaction mechanisms (single or double activation). The power-law model, together with quantitative experimental data, is an alternative to investigate the structure of a given pathway where the estimated value for the kinetic order is used to decide whether the process is mainly of single activation (*g *≈ 1), double activation (*g *≈ 2) or dual (intermediate values).

Finally, the consequences of this dual mechanism on the dynamics were investigated through the simulation of different experimental conditions. Towards this end, we simulated the behaviour of the system for two mutants: a mutant with a reduced number of receptors, and a second mutant in which one of the subunits of the receptors is blocked for the activation of the studied protein. Only in cases in which the system has the dual mechanism of activation both mutants would induce a different response, while a pure double protein activation system would not produce a significant response to the input signal, when one of the subunits of the homodimer receptors is constitutively blocked for the activation of the studied protein. Furthermore, our analysis suggests that in receptor-protein interacting systems with dual mechanism there may be an active switch between double and single activation in a way that intense double protein activation could initiate and dominate the signal in the short term (getting a fast intense signal), while single protein activation could control the system in the medium and long term (when input signal is weaker and decreases slowly).

Open questions that require attention relate to the underlying reasons that justify the dual activation mechanism. Mathematical modelling could be used to investigate why and how this dual mechanism is used by the cell to improve the performance of monomer-to-dimer transitions [5]. Moreover, there is increasing evidence for feedback inhibitor proteins that bind to activated receptors and compete with the true effectors for binding, e.g. CIS1, and Mig6 [32-34]. The expression of these feedback inhibitors is usually induced by the activated receptor. Thus, there may be an implicit switch between first and second order processes over the timecourse of stimulation dependent on feedback inhibitor levels that could be investigated in the next future.

## Authors' contributions

JV and TM designed the study, set up the mathematical model and performed the calculations concerning the responsiveness of the system under the supervision of OW Finally, all the authors including WK drafted the manuscript.

## Supplementary Material

Additional file 1 **On the role of receptor and protein homodimerisation in cell signalling Supplementary Material**. In the main text the double activation of the receptor is represented by a trimolecular biochemical reaction. The assumptions leading to this simplified representation of receptor activation are explained in detail. Furthermore, the determination of the apparent kinetic order using a power-law approach is discussed here. The supplementary material is provided as PDF-file. Click here for file
