# Supplementary material for: Dynamics of receptor and protein transducer homodimerisation
Source: BMC Syst Biol. 2008 Oct 31;2:92. doi: 10.1186/1752-0509-2-92 (PMC2650691; doi:10.1186/1752-0509-2-92)
Supplement: Additional file 1 — On the role of receptor and protein homodimerisation in cell signalling Supplementary Material. In the main text the double activation of the receptor is represented by a trimolecular biochemical reaction. The assumptions leading to this simplified representation of receptor activation are explained in detail. Furthermore, the determination of the apparent kinetic order using a power-law approach is discussed here. The supplementary material is provided as PDF-file. [file 1752-0509-2-92-S1.pdf]

Dynamics of receptor and protein transducer homodimerisation
Supplementary Material

Julio Vera [1], Thomas Millat[1], Walter Kolch[2,3] and Olaf Wolkenhauer*[1]

[1]University of Rostock, 18051 Rostock, Germany
[2]The Beatson Institute for Cancer Research, Glasgow G61 1BD, United Kingdom
[3]University of Glasgow, Sir Henry Wellcome Functional Genomics Facility, Glasgow, G12 8QQ, United Kingdom

Email: Olaf Wolkenhauer *ow@informatik.uni-rostock.de;

*Corresponding author

Abstract

Background: Signalling pathways are complex systems in which not only simple monomeric molecules interact, but also more complex structures that include constitutive or induced protein assemblies. In particular, the hetero- and homo-dimerisation of proteins is a commonly encountered motif in signalling pathways. Several authors have suggested in recent times that dimerisation relates to a series of physical and biological outcomes used by the cell in the regulation of signal transduction.

Results: In this paper we investigate the role of homodimerisation in receptor-protein interactions. Towards this end, mathematical modelling is used to analyse the features of such kind of interactions and to predict the behaviour of the system under different experimental conditions. A kinetic model in which the interaction between homodimers provokes a dual mechanism of activation (single and double protein activation at the same time) is proposed. In addition, we analyse under which conditions the use of a power-law representation for the system is useful. Furthermore, we investigate the dynamical consequences of this dual mechanism and compare the performance of the system in different simulated experimental conditions.

Conclusions: The analysis of our mathematical model suggests that in receptor-protein interacting systems with dual mechanism there may be a shift between double and single activation in a way that intense double protein activation could initiate and dominate the signal in the short term (getting a fast intense signal), while single protein activation could control the system in the medium and long term (when input signal is weaker and decreases slowly). Our investigation suggests that homodimerisation and oligomerisation are mechanisms used to enhance and regulate the dynamic properties of the initial steps in signalling pathways.

Two subsequent reactions as trimolecular elementary reaction

As described in detailed in the main article, we assume that the monomer P is activated via two different processes. Firstly, a monomer binds to the homodimeric receptor R which is described as a bimolecular reaction. However, because we are interested in the consequences with respect to the number of bound monomers we named this process single protein activation process. Note, that another classification scheme uses the number of simultaneous interactions as criterion. In this scheme a bimolecular reaction is also a first-order process.

Additionally, we assume that a second monomer P can bind to the other receptor subunit while a monomer is already bound to the first receptor subunit. As mentioned above we call such a process double protein activation process. Firstly, two monomers are bound simultaneously and secondly, a receptor interacts simultaneously with two monomers. The subsequent binding and consecutive activation of both monomers can be described as a sequence of two bimolecular reactions

$$\mathrm{R} + \mathrm{P} \quad \underset{c_2}{\overset{c_1}{\rightleftharpoons}} \quad \mathrm{RP} \tag{1a}$$

$$\mathrm{RP} + \mathrm{P} \quad \overset{c_3}{\longrightarrow} \quad 2\,\mathrm{P}^* + \mathrm{R}\,, \tag{1b}$$

where c_i denotes the rate coefficients to avoid confusions with the coefficients used in the main article. If the lifetime of the intermediary state RP is short the above mechanism can be presented approximately as a trimolecular reaction

$$2\,\mathrm{P} + \mathrm{R} \overset{k_3}{\longrightarrow} 2\,\mathrm{P}^* + \mathrm{R}\,. \tag{2}$$

However, with increasing lifetime of the intermediary state the complex structure of scheme (1) becomes more important and one has to use the detailed representation. However, the lifetime of the intermediary state RP is restricted by the first-order activation. Hence, we can assume that the double protein activation is well approximated by a formal trimolecular reaction. Furthermore, we focus on the changes to the apparent reaction order of a power-law representation as consequence of the existence of two parallel activation mechanism. The changing apparent order can be experimental evidence for such parallel processes. For an explicit analysis of a special homodimeric receptor one has to use a more detailed model.

Power-law modelling. Additional discussion

In order to illustrate the effect of using a power-law equation to describe the dual mechanism in the activation of the protein P, we investigated the value of the signal rate as function of the concentration of its inactive form P for different ratios of the kinetic parameters k_3/k_1. The results are shown in Figure 1(a-c). We calculated the value of the net signal rate represented as a power-law term

$$V(R^*, P) \approx \gamma_1 \cdot R^* \cdot P^g. \tag{3}$$

and compared it with the values obtained supposing that either a single or double protein activation term can represent an actual dual process. The apparent rate constant for the first and second approximation as well as the apparent rate constant and kinetic order for the power-law expression were calculated using the method described in [1] assuming an interval of feasible values for P in [0.0, 4.0].

For a low ratio k_3/k_1, the single activation approximation will agree with the original dual signal rate, but also the power-law representation fits the data assigning a value near to unity to the apparent kinetic order $g = 1.01$, Figure 1[a]. The double protein activation approximation provides a poor fitting of the original function. In case of a high value for this ratio, the situation is reversed. The double protein activation approximation produces a very good fit of the original data, while the single protein activation approximation is an inappropriate description. In this case, the agreement of the power-law representation is very good. In contrast to the previous situation, the apparent kinetic order is now almost two, $g = 1.99$ (Figure 1[c]). The most interesting situation is when intermediate values are considered for the ratio k_3/k_1.

In this case, only the power-law approximation gives an acceptable fitting of the data. The single and double protein activation approximations cannot reproduce the data, Figure 1[b].

The differences between the three approximations become evident if we investigate the dynamics of the considered homodimeric receptor-protein system and compare this to the mechanistic model (Figure 2). In this case the single activation approximation induces a higher but shorter peak of activation, while the double activation approximation induces a lower and flat peak of activation. Compared to this, the agreement between the original system and the power-law representation is much better than the other two cases.

Authors contributions

J.V. and T.M. designed the study, set up the mathematical model and performed the calculations concerning the responsiveness of the system under the supervision of O.W.. Finally, all the authors including W.K. drafted the manuscript.

Acknowledgements

The authors thank the collaboration of Taesoo Kwon and Ulf Schmitz elaborating a list of published homodimer receptor-homodimer transducer protein interactions used in initial versions of this work. This work was supported by the European Commission 6th Framework program and as part of the COSBICS project under contract LSHG-CT-2004-512060 (www.sbi.uni-rostock.de/cosbics) and by the German Federal Ministry of Education and Research (BMBF) through the projects BaCell-SysMo (0313978F) and COSMIC-SysMo (0313981D).

References

1. Hernandez-Bermejo B, Fairen V, Sorribas A: **Power-law modeling based on least-squares criteria: consequences for system analysis and simulation**. *Math. Biosci.* 2000, **167**:87–107.

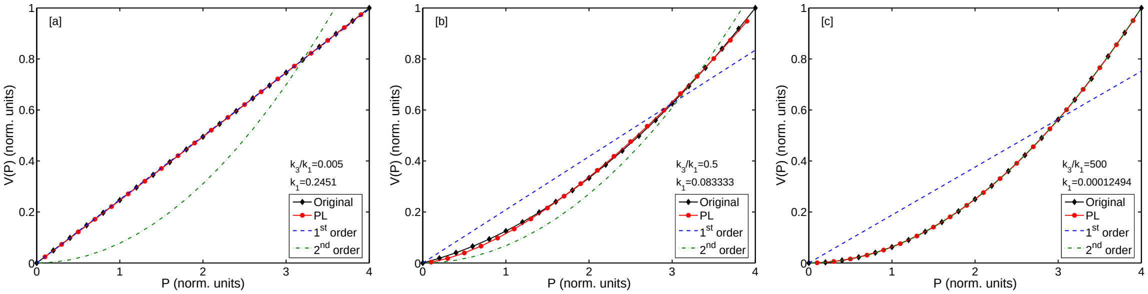

Figure 1: The net activation (3) for different ratios k_3/k_1 as function of the concentration of the inactive protein P. The original dual signal rate is represented with a black solid line and rhombs, the power-law approximation with a red solid line and circles, the single activation approximation with a blue dashed line and the double activation with a green dashed line. Three generic conditions for the ratio k_3/k_1 were analysed: low, medium and high values, and the value of k_1 was modulated to obtain a value of the function $V(P)$ in the same range $[0, 1]$, which facilitates the comparison of the three situations.

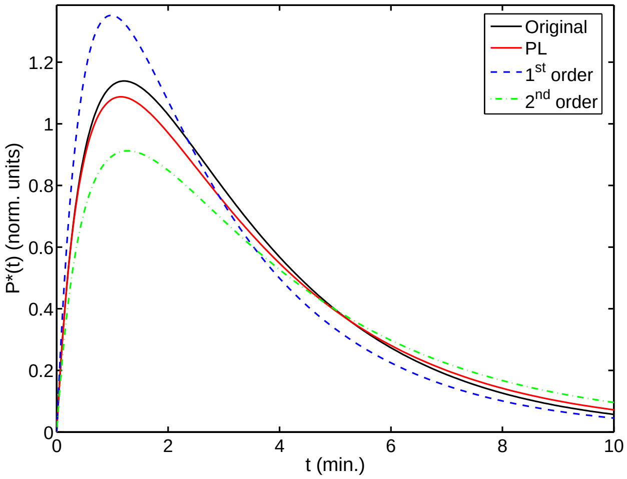

Figure 2: Dynamics of the activated protein P^* for the mechanistic model, the power-law representation and both single and double protein activation approximations. The values of the parameters are the same as in Figure 1 for the case of $k_3/k_1 = 0.5$. The initial state of the system was assumed as $P = 2.0, P^* = 0.0$. The stimulus of the system was simulated with a sustained value $R^*(t) = 5.0$.
